# Mastering the Art of Dermatosurgery: Aesthetic Alchemy in Medical Excellence

**DOI:** 10.7759/cureus.49659

**Published:** 2023-11-29

**Authors:** Osatohanmwen Ekomwereren, Abdullah Shehryar, Noor Abdullah Yahya, Abdur Rehman, Maryam Affaf, Srikar P Chilla, Uday Kumar, Nuzhat Faran, Mohammed Khaleel I.K.H. Almadhoun, Maria Quinn, Chukwuyem Ekhator

**Affiliations:** 1 Trauma and Orthopaedics, Royal Shrewsbury Hospital, Shrewsbury and Telford Hospital NHS Trust, Shrewsbury, GBR; 2 Internal Medicine, Allama Iqbal Medical College, Lahore, PAK; 3 Family Medicine, Dubai Medical College, Dubai, ARE; 4 Surgery, Mayo Hospital, Lahore, PAK; 5 Internal Medicine, Women’s Medical and Dental College, Abbotabad, PAK; 6 Medicine, CARE Hospitals, Hyderabad, IND; 7 School of Health Sciences, University of East London, London, GBR; 8 Medicine, Shaheed Mohtarma Benazir Bhutto Medical College Lyari, Karachi, PAK; 9 Internal Medicine, Fatima Memorial Hospital, Lahore, PAK; 10 Medicine and Surgery, Mutah University, Karak, JOR; 11 Internal Medicine, Jinnah Hospital, Lahore, PAK; 12 Neuro-oncology, New York Institute of Technology, College of Osteopathic Medicine, Old Westbury, USA

**Keywords:** patient outcomes, beauty enhancement, skin health, surgical innovations, modern techniques, ancient wisdom, evolution, medical advancements, aesthetic procedures, dermatosurgery

## Abstract

Dermatosurgery, a specialized branch within dermatology, has traversed an extraordinary journey through time, shaped by ancient practices, technological leaps, and shifting societal perceptions. This review explores the evolution of dermatosurgery, highlighting its profound transformation from addressing solely medical concerns to seamlessly integrating aesthetics. From its roots in ancient civilizations, where cultural traditions laid the foundation for modern techniques, to the twentieth-century technological renaissance, marked by innovative tools and enhanced understanding of skin anatomy, dermatosurgery has emerged as a dynamic field.

Societal notions of beauty and health have significantly influenced dermatosurgery, blurring the lines between medical necessity and elective aesthetic procedures. The delicate balance between satisfying aesthetic desires and upholding medical ethics is a central challenge that dermatosurgeons face today. Open dialogue between practitioners and patients as well as psychological support plays a pivotal role in navigating this terrain.

The training and ethics associated with dermatosurgery have evolved to meet the increasing demand for specialized procedures. Maintaining a focus on patient safety and satisfaction remains paramount as commercial pressures and disparities in access to care loom. Upholding best practices and standards in the field is essential for ensuring consistent, high-quality care for all patients.

Looking ahead, dermatosurgery stands on the brink of a transformative era, marked by non-invasive techniques, artificial intelligence (AI) integration, and personalized medicine. The field's ability to harmonize medical science with aesthetic artistry is evident in various case studies, showcasing the intricate balance dermatosurgeons strike between addressing medical concerns and fulfilling aesthetic desires. As dermatosurgery continues to evolve, it promises to provide patients with even more precise, tailored treatments that enhance both their physical well-being and aesthetic satisfaction.

## Introduction and background

Dermatosurgery, a specialized branch within dermatology, traces its roots back to ancient civilizations. The Ebers Papyrus, a medical document from ancient Egypt dating around 1500 BC, is one of the earliest known sources documenting skin ailments and treatments [1]. Over the centuries, the field has seen significant advancements, transitioning from merely addressing skin diseases to enhancing skin aesthetics.

The late nineteenth and early twentieth centuries marked a pivotal period in dermatosurgery. Major progress in local anesthesia during this era enabled dermatologists to perform biopsies and cutaneous surgeries with effective pain management for patients [2]. This period also witnessed a growing emphasis on the aesthetic outcomes of surgical procedures, reflecting societal values and the importance of physical appearance.

The blending of aesthetic principles with medical interventions has been a gradual process, influenced by both technological advancements and changing societal norms. Today, dermatosurgery not only addresses medical concerns but also places a strong emphasis on achieving aesthetically pleasing results. Procedures such as dermabrasion, laser surgeries, and filler injections, while medically sound, are also designed to cater to the increasing societal emphasis on beauty and youthful appearance [3].

Dermatosurgery occupies a unique position among surgical disciplines. The majority of surgical interventions in this field can be performed under local or regional anesthesia, often in smaller procedure rooms separated from larger operating suites [4]. This flexibility, combined with the field's focus on aesthetics, underscores the harmonization of medical efficacy with aesthetic outcomes.

As dermatosurgery continues to evolve, it offers a holistic approach to patient care, where the goal is not just medical wellness but also psychological contentment and aesthetic satisfaction. The promise of dermatosurgery lies in its ability to seamlessly integrate the science of medicine with the art of aesthetics, offering patients comprehensive care that addresses both their medical and aesthetic needs [5].

## Review

The evolution of dermatosurgery: bridging ancient wisdom with modern advancements

Dermatosurgery, a distinctive subspecialty nestled within the broader realm of dermatology, has embarked on an extraordinary journey through the annals of time. Its trajectory is marked by a captivating interplay between ancient wisdom, state-of-the-art technologies, and the ever-fluctuating currents of societal values. This profound evolution stands as a testament to the enduring human spirit of ingenuity as well as the relentless pursuit of not only physical health but also the timeless aspiration for aesthetic beauty.

Ancient Practices and Traditional Techniques

The origins of dermatosurgery delve deep into the annals of ancient civilizations. Historical treasures like the revered Ebers Papyrus, dating back to ancient Egypt, offer us precious glimpses into early skin treatments [6]. These age-old techniques, profoundly influenced by cultural customs and traditions, served a multitude of purposes, from addressing skin ailments to excising tumors and even enhancing beauty. While they may appear rudimentary by today's standards, these practices laid the foundational groundwork for the contemporary dermatological procedures we know today.

Technological Breakthroughs and Their Implications

The twentieth century ushered in an era of unprecedented technological advancement in dermatosurgery. Meticulously crafted surgical instruments, coupled with a deepened understanding of the intricacies of skin structure, marked a turning point in this field. Pioneering procedures like laser surgeries, dermabrasion, and the skillful application of filler injections rose to prominence, epitomizing precision, efficacy, and minimally invasive approaches [7]. The meteoric rise of dermatosurgery in recent decades is a testament to the growing demand for skilled dermatosurgeons, underscored by the increasing prevalence of skin malignancies and the advent of intricate operations such as sentinel lymph node biopsies [8].

The Oscillating Societal Paradigms of Beauty and Wellness

The evolution of dermatosurgery is intimately intertwined with society's ever-evolving notions of beauty and well-being. The Renaissance, an era celebrated for its appreciation of the human form and aesthetics, catalyzed the infusion of artistry into medical endeavors [9]. In today's world, the line between aesthetic enhancements and therapeutic interventions has become increasingly blurred. Dermatosurgery now stands at the crossroads of medical pragmatism and societal aesthetics, responding to an amplified emphasis on radiant beauty, ageless vitality, and holistic health. Notably, the last four decades have been pivotal in catalyzing paradigm shifts in dermatosurgery, shaping innovative treatments, and enhancing the overall patient experience [10].

The science behind aesthetics in dermatosurgery

The skin, an intricate tapestry that envelops our body, stands as the body's most expansive organ. More than just a protective barrier, it's a labyrinth of complexity, weaving together layers, cells, and functions. Each layer, from the surface epidermis to the deeper dermis and the subcutaneous tissue below, boasts distinct attributes that are foundational to the outcomes of dermatological interventions [11].

Central to dermatosurgery's success is an understanding of the multifaceted criteria that constitute societal perceptions of beauty. Historically, the ideals of beauty have been influenced by notions of proportions, harmony, and specific attributes. The golden ratio, a revered mathematical relationship approximating 1 to 1.618, has left its indelible mark on art, architecture, and nature. In the realm of human aesthetics, it is often postulated that this ratio delineates the proportions of an "ideal" face - from the positioning of the eyes to the curve of the lips [12]. In dermatosurgery, this conceptualization is not merely theoretical. It provides a framework, a guiding beacon, directing surgeons as they endeavor to either accentuate or recapture these idealized proportions.

But dermatosurgery's scope transcends the mere remediation of skin anomalies. It is a discipline that elegantly intertwines the rigors of medical science with the nuances of aesthetic artistry. Every incision, excision, or intervention carries dual objectives: addressing the medical concern while remaining acutely conscious of aesthetic outcomes. Be it the subtle art of minimizing scars, the finesse required in contouring facial features, or the precision in restoring lost volume, dermatological procedures aim for an equilibrium - results that not only stand up to medical scrutiny but also resonate with the patient's aesthetic aspirations [13]. In essence, dermatosurgery is both science and art. Its practitioners are tasked with the responsibility of ensuring health and healing, all while crafting outcomes that align with the ever-evolving definitions of beauty.

Core dermatosurgical procedures: a synergy of aesthetics and medicine

The realm of dermatosurgery encompasses an intriguing fusion of aesthetics and medicine, wherein various procedures are meticulously designed to not only address medical concerns but also prioritize aesthetic outcomes. These procedures, driven by the quest for both health and beauty, have ushered in a new era of dermatological treatments.

One such example is Mohs surgery, an intricately precise surgical technique employed in the treatment of skin cancer. In this procedure, the cancerous tissues are methodically excised layer by layer, and each layer is examined during the surgery. This meticulous approach ensures that minimal damage is inflicted upon the surrounding healthy tissue, ultimately resulting in superior cosmetic outcomes [14].

Scar revision procedures, too, are guided by the principle of minimalism, seeking to enhance the aesthetic appeal of scars. Dermatologists employ an array of techniques, including dermabrasion, laser therapy, and surgical revision, to render scars less conspicuous. By skillfully implementing these methods, dermatosurgeons aim to restore a more refined appearance to the affected skin [15].

Hair transplant surgery, while primarily addressing functional concerns related to hair loss, also places significant emphasis on aesthetics. The procedure involves the delicate transplantation of hair follicles from one region of the body to another. This dual-purpose intervention not only combats hair loss but also ensures that the results are characterized by a natural and aesthetically pleasing appearance [16].

In the realm of dermatosurgery, lasers have heralded a revolution. These high-precision tools have transcended the confines of traditional medicine, permeating the realm of cosmetic enhancement. Lasers offer the unique ability to provide targeted, precise treatments, making them indispensable in fine-tuning skin health and beauty. Whether employed to remove blemishes, reduce wrinkles, or enhance skin tone, lasers play an instrumental role in the pursuit of aesthetic perfection [5].

The role of patient expectations

The realm of dermatosurgery has witnessed significant advancements over the years, with a focus on not just treating skin conditions but also enhancing aesthetic appeal. As the field evolves, understanding and managing patient expectations have become paramount. This is especially true given the delicate balance between aesthetic desires and medical necessities, the dialogue between dermatosurgeons and patients, and the psychological implications of undergoing dermatosurgical procedures.

The Balance Between Aesthetic Desires and Medical Necessities

Dermatosurgery, at its core, merges the principles of aesthetics with medical interventions. While patients often seek procedures to enhance their appearance, there is a medical underpinning to every intervention. For instance, while a patient might desire the removal of a mole for cosmetic reasons, the dermatosurgeon must also consider the medical implications, such as the potential for malignancy. This balance is a fine line for physicians to tread, ensuring that while aesthetic desires are met, medical necessities are not compromised [17].

Managing Expectations: The Dialogue Between Dermatosurgeon and Patient

Patient satisfaction in dermatologic surgery is closely tied to the alignment of expectations between the physician and the patient. Dermatosurgeons need to have open dialogues with their patients, understand their desires, and set realistic expectations. This involves explaining the potential outcomes, risks, and benefits of the procedure. By ensuring that patients have a clear understanding of what to expect, dermatosurgeons can enhance patient satisfaction and reduce the likelihood of postoperative dissatisfaction [9].

Psychological Impacts and the Importance of Pre-surgery Counseling

Anticipating surgery can be a source of emotional distress for many patients [18]. Concerns may range from fears about the procedure, potential complications, recovery process, and even interactions with medical staff. Dermatosurgeons must recognize these concerns and address them through pre-surgery counseling. Psychological interventions, such as relaxation techniques, cognitive-behavioral therapies, and coping strategies, have been shown to reduce anxiety and improve surgical outcomes [19]. By addressing the psychological aspects, dermatosurgeons can ensure a holistic approach to patient care, enhancing both the physical and mental well-being of their patients.

Training and ethics in dermatosurgery

Dermatosurgery, a specialized field within dermatology, has witnessed significant advancements over the years. As the field continues to evolve, the training and ethical considerations associated with it have become paramount. This section delves into the current curriculum for aspiring dermatosurgeons, the ethical challenges faced, and the best practices upheld in the field.

The Evolving Curriculum for Aspiring Dermatosurgeons

The journey of dermatosurgery education has been transformative. Historically, the focus was primarily on general dermatology. However, with the increasing demand for specialized procedures, the curriculum has expanded to encompass a wider range of surgical techniques. The American Academy of Dermatology, for instance, offers a basic dermatology curriculum that includes modules on general dermatology tailored for various specialties, from internists to pediatricians [20]. As the field advances, the curriculum needs to be regularly updated, ensuring that budding dermatosurgeons are equipped with the latest knowledge and skills.

The Ethical Dimensions: Navigating Commercial Pressures and Patient Well-Being

Dermatosurgery, like many medical specialties, is not immune to ethical dilemmas. The convergence of aesthetics and medicine often brings commercial pressures to the forefront. With the rising demand for cosmetic procedures, there is a temptation to prioritize profit over patient well-being [21]. Dermatosurgeons must maintain a balance, ensuring that patient safety and satisfaction remain at the core of their practice. Ethical considerations also extend to managing patient expectations, especially in a field where outcomes can significantly impact self-esteem and psychological well-being [22].

Best Practices and Standards in the Field

Upholding best practices in dermatosurgery is essential for ensuring patient safety and achieving optimal outcomes. Procedures like Mohs surgery, scar revisions, and laser treatments require a high degree of precision and expertise. Adhering to established guidelines and continuously updating one's skills through professional development is vital. Furthermore, with the increasing popularity of dermatological procedures, there is a need for standardized protocols and guidelines to ensure consistency in treatment and care across the board.

Challenges and controversies in dermatosurgery

Dermatosurgery, much like any specialized medical field, confronts its fair share of challenges and controversies. Throughout its evolution, this discipline has been scrutinized both by the medical community and the general public. In this section, we explore some of the most pressing issues facing dermatosurgery today.

Potential Risks and Complications

Every surgical procedure inherently carries certain risks, and dermatosurgery is no exception. While many of these procedures are considered minimally invasive, complications can and do occur. These complications encompass a broad spectrum, ranging from relatively minor issues such as infections, scarring, and pigmentary changes to more serious and potentially severe problems like nerve damage or adverse reactions to anesthesia [23].

For both patients and practitioners, understanding and addressing these risks are of paramount importance. Patients must be thoroughly informed about the potential complications associated with dermatosurgery procedures, ensuring they can make informed decisions about their care. Conversely, dermatosurgeons must continually refine their techniques and prioritize meticulous postoperative care to minimize the likelihood of complications and optimize patient outcomes. The effective management of risks and complications remains an ongoing challenge within the field of dermatosurgery, necessitating constant vigilance, innovation, and a commitment to delivering the highest standard of care.

The debate over "unnecessary" aesthetic procedures in dermatosurgery

In the realm of dermatosurgery, the distinction between essential medical interventions and elective aesthetic procedures can often become muddled. There is an ongoing discourse surrounding the contention that some procedures are driven more by societal beauty standards than genuine medical necessity [24]. For instance, the practice of dermatologists dispensing nonprescription skin products and the performance of certain cosmetic procedures have become subjects of debate within the profession [25]. While these procedures can undeniably boost self-esteem and overall well-being, it is imperative to weigh the potential risks against the benefits.

Addressing Disparities and Access to Dermatological Care

Equitable access to high-quality dermatological care is far from uniform across different populations. Factors such as socioeconomic status, geographic location, and even racial background can significantly influence the level of care individuals receive [26]. Teledermatology, a burgeoning practice, has emerged as a promising solution to bridge disparities in care, especially in the context of the COVID-19 pandemic [27]. Nonetheless, more concerted efforts are required to ensure that everyone, regardless of their background, enjoys equal access to the finest dermatological treatments available.

Dermatosurgery undeniably offers promising solutions to a wide range of skin-related issues, so it is imperative to approach this field with a discerning perspective. By confronting these challenges head-on and fostering open dialogue between patients and healthcare providers, dermatosurgery can continue to evolve in a manner that prioritizes not only aesthetic outcomes but also the holistic well-being of patients.

Case studies: balancing aesthetics and medicine in dermatosurgery

The realm of dermatosurgery is a captivating fusion of medical science and artistic finesse. Over the years, dermatosurgeons have encountered a diverse array of cases, each posing its own set of challenges and opportunities. Here, we delve into a series of case studies that exemplify the delicate equilibrium between aesthetics and medicine in various scenarios.

Case Study 1: Acne Scar Revision

A 28-year-old female patient sought dermatological assistance for the significant post-acne atrophic scars marred across her cheeks and forehead. Though her acne had been medically treated years ago, the lingering scars continued to inflict a heavy toll on her self-esteem [28]. The dermatosurgeon devised a comprehensive strategy that combined subcision, dermal fillers, and laser resurfacing. The outcome was not only a substantial improvement in the texture of her skin, effectively addressing the scars, but also an enhancement of her overall facial aesthetics. This transformation not only mended the physical scars but also rejuvenated her self-confidence.

Case Study 2: Rhinophyma Treatment

A 55-year-old male patient presented with rhinophyma, a condition characterized by an enlarged, bulbous, and conspicuously red nose [29]. Beyond the medical concerns associated with this condition, the patient was profoundly distressed by the altered appearance of his nose. Employing a surgical approach that melded scalpel and laser techniques, the dermatosurgeon meticulously reshaped the nose. This intervention not only remedied the medical aspects of the condition but also aligned with the patient's aesthetic aspirations. It offered him a renewed sense of confidence and contentment, transcending the boundary between medical necessity and aesthetic desire.

These case studies underscore the intricate tightrope dermatosurgeons walk as they navigate the intricate nexus of medical necessities and aesthetic aspirations. Each patient presents a unique narrative, and the artistry of dermatosurgery lies in crafting solutions that seamlessly harmonize both facets, medicine and aesthetics, to ultimately restore both physical health and emotional well-being.

Future prospects and innovations in dermatosurgery

Dermatosurgery, like many other medical fields, is on the cusp of a transformative era, driven by technological advancements and a deeper understanding of human biology. The future promises procedures that are less invasive, more precise, and tailored to individual needs. Following is a glimpse into the innovations shaping the future of dermatosurgery.

The Rise of Non-invasive and Minimally Invasive Techniques

The demand for non-invasive and minimally invasive procedures is on the rise, driven by patients' desire for quicker recovery times, fewer scars, and reduced risk of complications. Techniques such as radiofrequency, ultrasound, and cryolipolysis are revolutionizing the way dermatosurgeons approach skin treatments, offering effective results with minimal downtime [30].

*Integration of Artificial Intelligence* *and Robotics in Dermatosurgery*

Artificial intelligence (AI) is gradually changing the landscape of surgery, with advancements in imaging, navigation, and robotic intervention [31]. In dermatosurgery, AI can assist in diagnosing skin conditions, predicting treatment outcomes, and even guiding surgical procedures in real-time. Robotic systems, equipped with AI algorithms, can enhance precision, reduce human error, and potentially improve patient outcomes [32]. The integration of AI in surgical robotics holds the promise of procedures that are not only more accurate but also more consistent [33].

Personalized Dermatosurgery

The concept of personalized medicine is reshaping healthcare, emphasizing treatments tailored to individual genetic makeup and features [34]. In dermatosurgery, this means procedures that consider a patient's unique genomic profile, skin type, and aesthetic preferences. Understanding the genetic factors that influence skin aging, wound healing, and response to treatments can lead to more effective and tailored interventions [35]. The future may see dermatological procedures that are not only personalized based on genetic makeup but also on individual aesthetic goals and needs.

## Conclusions

Dermatosurgery marries the advances of medical science with society's evolving aesthetic aspirations. From its ancient foundations to modern technological innovations, it caters to a spectrum of patient needs, blending therapeutic remedies with aesthetic finesse. As the field undergoes rapid transformations with innovations like non-invasive techniques, AI integration, and personalized approaches, it is crucial to address associated challenges, ensuring patient safety and equitable access to care. At its core, dermatosurgery converges the realms of art and science, offering treatments that rejuvenate both body and spirit. Looking ahead, its potential to enhance lives globally is both vast and exhilarating.
